# Novel perspective on field recordings in zebrafish models of epilepsy

**DOI:** 10.1186/1471-2202-16-S1-P171

**Published:** 2015-12-18

**Authors:** Adriana Dabacan, Sorana Ciura, Edor Kabashi, Hortense de Calbiac, Raul Muresan

**Affiliations:** 1 Coneural, Romanian Institute of Science and Technology, Cluj Napoca, Romania; 2 Basis of Electronics, UTCN, Cluj-Napoca, Romania; 3 Amyotrophic Lateral Sclerosis: From Genetics to Treatment, ICM, Paris, France

## 

Research in Epilepsy relies strongly on animal models, either for describing genetic conditions involved in the disease or for testing potential drugs that might alleviate the symptoms [1].

In this study, we looked at the differential effect of Pentylenetetrazole (PTZ), a well-known epileptogenic drug [2], on 5-6 dpf zebrafish larvae of two different genetic conditions: a mutant line, where a gene known to be involved in focal epilepsy was specifically knocked down, as well as control mismatch oligonucleotide-injected zebrafish.

The zebrafish larvae were scored for phenotypic features, including hyperactivity and aberrant locomotion. These phenotypic features were present in all mutant fish recorded, but were absent in the mismatch control. We acquired electrophysiological field recordings from the optic tectum before and after PTZ application and selected a low frequency band of the signal (0.05 - 0.5 Hz). Troughs, representing epileptic events, were extracted and a wide range of analyses were applied: total event count, event time histogram, event duration distributions, inter-event-interval (IEI) distribution, classical and scaled autocorrelations on the field and event signals [3].

Traditional analysis provided a quantitative evaluation of PTZ-induced epileptic events: an increase in number of epileptic events was observed in mutant fish with respect to control, and the event time histogram showed a more abrupt increase at 32-37 min. after PTZ application, whereas in the control case, the increase was constant throughout the response period. Compared to controls, in mutant fish, PTZ application led to a larger increase in the number of short (0.5-1.5 s) events with small IEI (< 10s).

**Figure 1:**
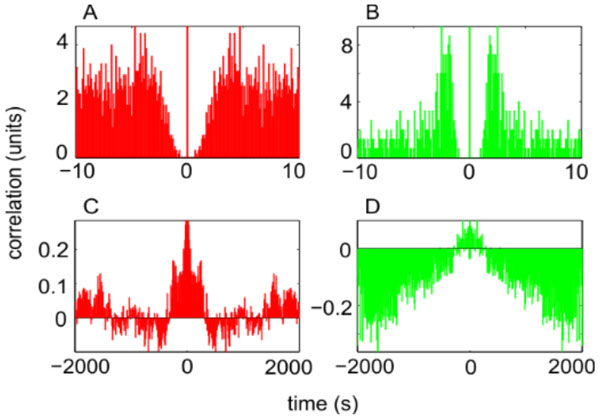
**Correlation histograms on events from zebrafish field recordings: Top: Autocorrelation of mutant (A) versus control (B) animals; Bottom: Scaled autocorrelation with scale segment of 1000s of mutant (C) versus control (D) animals**.

Correlation analysis revealed qualitative information about the effect of PTZ on field recordings: The scaled autocorrelation (scale segment of 1s) on the field signal exhibited oscillatory components around 0.4 Hz in all conditions, but mutant fish exhibited frequency variability with time after PTZ application and variability across animals, leading to a low average oscillation power. In controls the frequency was robustly locked at 0.4 Hz. Autocorrelation histograms computed on the extracted events evidenced a wide refractory period (~ 4 s, see Figure 1A), followed by baseline in mutant fish recordings, whereas control animals exhibited a narrow refractory period (~ 2 s, see Figure 1B), followed by a secondary peak and a slow modulation (~ 35 s). Scaled autocorrelation histograms (scale segment of 1000s) showed constant correlation decrease with increased lag in the control fish, but not in mutant fish, where correlation for larger lags fluctuated around zero (see Figure 1C and 1D).

The characteristics revealed by correlation analysis suggest potentially different mechanisms underlying PTZ-induced epileptic events. We therefore propose that, in addition to the traditional statistics on epileptic events, looking at the temporal characteristics and correlation structure of field recordings may lead to a better classification and understanding of the mechanisms underlying epileptogenesis in various models of epilepsy.
